# Characterization of Fatty Acids in Crenarchaeota by GC-MS and NMR

**DOI:** 10.1155/2015/472726

**Published:** 2015-12-31

**Authors:** Timothy Hamerly, Brian Tripet, Louie Wurch, Robert L. Hettich, Mircea Podar, Brian Bothner, Valérie Copié

**Affiliations:** ^1^Department of Chemistry and Biochemistry, Montana State University, Bozeman, MT 59717, USA; ^2^Oak Ridge National Laboratory, Oak Ridge, TN 37831, USA; ^3^Department of Microbiology, University of Tennessee, Knoxville, TN 37996, USA; ^4^Thermal Biology Institute, Montana State University, Bozeman, MT 59717, USA

## Abstract

Lipids composed of condensed isoprenyl units connected to glycerol backbones by ether linkages are a distinguishing feature of Archaea. Data suggesting that fatty acids with linear hydrocarbon chains are present in some Archaea have been available for decades. However, lack of genomic and biochemical evidence for the metabolic machinery required to synthesize and degrade fatty acids has left the field unclear on this potentially significant biochemical aspect. Because lipids are energy currency and cell signaling molecules, their presence in Archaea is significant for understanding archaeal biology. A recent large-scale bioinformatics analysis reignited the debate as to the importance of fatty acids in Archaea by presenting genetic evidence for the presence of enzymes required for anabolic and catabolic fatty acid metabolism across the archaeal domain. Here, we present direct biochemical evidence from gas chromatography-mass spectrometry (GC-MS) and nuclear magnetic resonance (NMR) spectroscopy for the presence of fatty acids in two members of the Crenarchaeota,* Sulfolobus solfataricus* and* Ignicoccus hospitalis*. This is the first report providing biochemical data for the existence of fatty acids in these Crenarchaeota, opening new discussions on energy balance and the potential for the discovery of new thermostable enzymes for industry.

## 1. Introduction

Fatty acids have two distinct biological roles. On the one hand, they are energy-rich molecules, and on the other hand, they play an important role as structural components of membranes of bacterial and eukaryotic cells [1–6]. As the most reduced form of carbon, saturated fatty acids are high-energy molecules, yet sufficiently stable to be stored for long periods of time with minimal degradation [2, 4–6]. Fatty acids undergo *β*-oxidation, which converts long chain fatty acids into two-carbon acyl-coenzyme A (acetyl-CoA) molecules, which are then used in the citric acid cycle for energy production [7, 8]. Fatty acids are also membrane components of prokaryotic and eukaryotic cells, playing an important role in cell structure and integrity.

**Figure 1:**
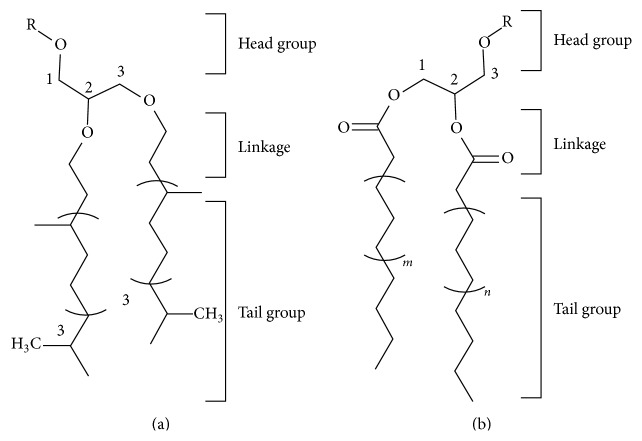
Structural representation of characteristic lipids used in archaeal and Prokaryotic/Eukaryota membranes. (a) Structure of a basic isoprenoid-based lipid used in archaeal membranes. (b) Canonical acyl chain-based lipid used in the formation of Prokaryotic/Eukaryota membranes. For each lipid, a head group consisting of any number of R groups connected to a glycerol molecule is depicted. Also attached to the glycerol head group are two hydrocarbon-based chains. The three major differences between the two types of lipids are in the chemical linkage to glycerol, the position on glycerol where the linkage takes place, and the type of hydrocarbon chain present. For archaeal lipids, five carbon isoprenoid units with a methyl group branched at every fourth carbon and are attached by ether linkages to glycerol at the 2 and 3 positions. For Prokaryotic/Eukaryota, hydrocarbon chains (*m* and* n* in (b)) vary in length from 12 to 26 and are linked by ester bonds at the 1 and 2 positions to glycerol and not typically branched.

The discovery of Archaea as the third domain of life in the early 1970s revealed a new class of lipids based on isoprenoid moieties [9–11]. These isoprenoid lipids were found to have 20 (archaeol) or 40 (caldarchaeol) carbons often linked as a cyclic molecule (Figure 1) [12]. Instead of linear carbon chains like the majority of those found in prokaryotic and eukaryotic cells, the archaeal lipids are branched every fourth carbon, with a single methyl group linked to each of these carbons. Archaeal lipids also differ in their linkage to glycerol, having an ether linkage as opposed to an ester linkage, and are thus termed fatty ether lipids [12]. The ether linkage is crucial for the stability of these lipids in the high temperature environments where many of the first archaeal organisms were first isolated and characterized. These fatty ether linkages have been subsequently found to exist in plants, covalently attached to chlorophyll, and as components of the platelet activating factor in mammals [13–15]. Ether-linked lipids can assemble as either a lipid bilayer with two archaeol molecules or are cyclized to form tetraether lipids with two glycerol molecules comprising the entire lipid membrane [12, 16]. The linkage of the two chains also differs with respect to the carbon position of the glycerol scaffold to which the two chains are linked: archaeal lipids have the chains attached onto the 1st and 2nd carbon positions, while prokaryotic/eukaryotic lipids have the chains linked to the 2nd and 3rd positions of the glycerol backbone (Figure 1) [3, 17].

Until recently, it was thought that most Archaea possessed only ether-linked (fatty alcohol) lipids. The ether bond has greater stability at high temperature than an ester bond, and the protein machinery for synthesis and breakdown of fatty acids had not been identified [18–20]. Studies of the hyperthermophilic euryarchaeon* Pyrococcus furiosus* identified 18 fatty acids, including saturated, monounsaturated, and dicarboxylic fatty acids [20]. However, general support for this finding across archaeal species in the form of wide spread genome annotation is only now becoming available. Genomic analysis of nearly 70 archaeal organisms revealed genes with sequences similar to those coding for enzymes capable of carrying out fatty acid metabolism in bacteria and eukaryotes [21]. Using bacterial *β*-oxidation as a model, it was revealed that four enzymes are needed for fatty acid metabolism, and subsequent searches of archaeal genomes using the COG database revealed genes encoding enzymes for *β*-oxidation within archaeal organisms. Two organisms whose genomes were analyzed by Dibrova et al. (*Ignicoccus hospitalis* and* Sulfolobus solfataricus*) have been previously investigated at functional genomics levels, as model extremophiles for understanding archaeal biology and response to stress [21–25].* I. hospitalis* has been of interest due to its minimalistic genome and its unusual interaction with another archaeon,* Nanoarchaeum equitans* [24–28]. It has been postulated that* S. solfataricus* contains multiple enzymes for each step necessary for fatty acid metabolism, making this archaeon an excellent target for fatty acid analysis. On the other hand, the minimalistic genome of* I. hospitalis* has been predicted to lack some of the requisite machinery for fatty acid *β*-oxidation, having genes encoding enzymes to carry out three of the catabolic steps, but lacking a predicted homologue of the acyl-coenzyme A (CoA) dehydrogenase which initiates *β*-oxidation [21]. Dibrova et al. speculated that a distant homolog of acyl-CoA dehydrogenase (ACD) may carry out the initiation reaction in* I. hospitalis*, further stating that this gene is proximal to other *β*-oxidation genes [21]. Additionally, it was reported by Jahn et al. that* I. hospitalis* could coutilize acetate for energy, providing evidence that catabolism of fatty acids could be used as an alternative energy source [29]. Nevertheless, the authors were unable to confirm if acetate actually plays a role in fatty acid metabolism [21]. With genomic evidence suggesting the presence of enzymes necessary for fatty acid metabolism, we set out to investigate their presence in two archaeal organisms using* in vitro* biochemical approaches.

In this study, a combination of gas chromatography-coupled mass spectrometry (GC-MS) and solution nuclear magnetic resonance spectroscopy (NMR) was used to analyze the fatty acid content of two hyperthermophilic archaeal organisms. Both organisms are members of the phylum Crenarchaeota but differ from one another in that* S. solfataricus* grows aerobically, while* I. hospitalis* is a strict anaerobe living in a highly reducing environment, which would suggest that fatty acid *β*-oxidation is a relatively costly energetic commitment for this organism. Nevertheless, the process of *β*-oxidation in a reducing environment might be possible considering that other hyperthermophiles such as* Archaeoglobus fulgidus* couple fatty acid *β*-oxidation with sulfate reduction [30]. The presence of fatty acids was confirmed in both* I. hospitalis* and* S. solfataricus*, providing new insights into archaeal biology and into how energy in the form of fatty acids may be stored and used in these microbes. The confirmation of the fact that these archaeal organisms contain fatty acids suggests novel gene architecture to support fatty acid metabolism in Archaea and has implications for biotechnology.

## 2. Materials and Methods

### 2.1. Materials

All solvents for metabolite extraction were purchased as HPLC grade, including methanol, acetone, and hexanes from EMD Chemicals Inc. (Gibbstown, NJ), and chloroform from Avantor (Center Valley, PA). BF_3_ in methanol (Supelco 33020-U) for derivatization of fatty acids was purchased from Sigma-Aldrich (St. Louis, MO). A mixture of fatty acid standards (GLC 538) used for GC-MS and NMR analysis was purchased from Nu-Chek Prep Inc. (Elysian, MN). DSS (4,4-dimethyl-4-silapentane-1-sulfonic acid) used for NMR chemical shift referencing and metabolite quantification was purchased from Sigma-Aldrich (St. Louis, MO). All solvents were used as supplied without further purification.

### 2.2. Cell Culture

*S. solfataricus* (P2) was prepared in batch cultures as previously described [24]. Briefly,* cells* were grown aerobically in DSMZ 182 media, with the addition of 0.1% glucose, at pH 3, and 80°C. For these experiments, 50 mL of cell culture was collected at late-log phase, for maximum cell density. Cells were cooled in liquid nitrogen to just above freezing prior to pelleting by centrifugation (20,000 ×g for 15 minutes) and stored at −80°C until analysis.

Cultures of* I. hospitalis* and* I. hospitalis*-*N. equitans* were prepared as previously described [27, 31, 32]. Briefly, cultures were grown for 24 hours in 1 L bottles containing 250 mL 0.5x SME medium, sulfur (10 g/L), and a H_2_-CO_2_ (80–20%) gas phase (15 psi), at 85°C. Cell cultures were cooled to room temperature before harvesting and then chilled further on ice, and, finally, cells were collected by centrifugation (8000 ×g for 20 minutes). The cell pellets were washed with cold, anaerobic 0.5x SME medium, aliquoted in Eppendorf tubes, flash-frozen with liquid nitrogen under N_2_ gas, and stored at −80°C until analysis.

### 2.3. Extraction of Fatty Acids

Fatty acids were extracted using a modified Bligh-Dyer method [33]. Briefly, cell pellets were resuspended in phosphate buffer saline (PBS) and transferred to glass vials. Cells were pelleted by centrifugation at 2000 ×g. Following centrifugation, PBS was removed, and a volume of methanol (MeOH) was added equal to three times that of the cell pellet. Cells were lysed using a tissue homogenizer, followed by addition of chloroform (CHCl_3_) at an equal volume to that of MeOH. The resulting solution was placed in a vortex mixer at +4°C and shaken slowly for 45 minutes. Cell debris was pelleted by centrifugation at 2000 ×g and the resulting supernatant transferred to a new glass vial. A wash solution of MeOH/CHCl_3_ was added to the cell debris, vortex briefly pelleted as before, and resulting liquid combined with the previously collected supernatant. To this solution, cold acetone was added and the vials were left at −80°C overnight to precipitate proteins. Precipitated proteins were removed by centrifugation at 2000 ×g and supernatant transferred to another glass vial and dried down under N_2_ for storage. Samples were kept at −80°C until analysis by GC-MS and NMR.

### 2.4. Preparation of Fatty Acid Methyl Esters (FAMES)

Metabolites extracted from cells and fatty acid standard mixture in hexanes were transferred to 2 mL glass GC vials and dried under N_2_. To each vial, 400 *μ*L of BF_3_-MeOH was added. The solution was heated at 60°C for 15 minutes to facilitate the esterification of fatty acids. The reaction was quenched by the addition of 200 *μ*L of H_2_O, followed by 200 *μ*L of saturated NaCl solution and 500 *μ*L of hexanes. Vials were vortexed and then left to stand for 15 minutes until solvent layers were clearly separated. The top layer comprised of hexanes and hydrophobic molecules was transferred to a new glass GC vial. A second extraction of the saturated NaCl solution was performed using an additional 500 *μ*L of hexanes and combined with the original 500 *μ*L fraction. This extraction procedure ensured the maximum recovery of fatty acid methyl ester derivatives (FAMES). Lastly, the resulting FAMES solution was dried under N_2_ gas, before being resuspended in 100 *μ*L of hexanes and transferred to a 150 *μ*L glass GC insert for GC-MS analysis.

### 2.5. GC-MS Data Acquisition and Analysis

FAMES were analyzed on an Agilent 7890A GC-MS equipped with an Agilent 7693 Autosampler and an Agilent 5975C inert XL EI/CI MSD with Triple-Axis Detector (Santa Clara, CA), operated using the Agilent MSD ChemStation software (Santa Clara, CA). A Zebron ZB-FFAP column with a length of 60 meters, inner diameter of 0.25 mm, and film thickness (nitroterephthalic acid modified polyethylene glycol phase) of 0.25 *μ*m was used for separation (Phenomonex, Torrance, CA). Parameters for acquisition were as follows: 1 *μ*L of sample was injected into a SGE-092010 split/splitless liner (Austin, Texas) with an inlet temperature of 250°C and helium gas flow of 62.5 mL per minute. The GC oven contained a 5-meter fused silica guard column, connected to the analytical column for a total length of 65 meters. Temperatures of the GC oven throughout the run were as follows: initial temperature of 120°C, held for 4 min, a ramp in temperature of 6.5°C per minute to 170°C, then a ramp of 2.75°C per minute to 250°C held for 9 minutes, for a total run time of 50 minutes. A solvent delay of 4 minutes was set before MS acquisition began. The transfer line from GC column to MS was set to 250°C, the source 230°C, and the quadrupole 150°C. Source fragmentation was done by electron ionization (EI) at 70 eV, with a scan range of 35 amu to 450 amu (atomic mass units), and scan rate of 1.80 scans per second. Data was visualized using the Agilent Mass Hunter Workstation Qualitative Analysis software. Matches for fatty acids were confirmed manually and by searching the NIST database using the NIST Mass Spectral Search Program [34].

### 2.6. NMR Data Acquisition and Analysis

For ^1^H 1D NMR analysis, metabolite extracts and fatty acid standard mix (see above) were resuspended in 500 *μ*L of deuterated chloroform (CDCl_3_) and transferred to 5 mm NMR tubes. NMR spectra were acquired using a 600 MHz (^1^H Larmor frequency) AVANCE III solution NMR spectrometer from Bruker Daltonics (Billerica, MA) equipped with a 5 mm triple resonance (^1^H, ^15^N, ^13^C) helium-cooled TCI cryoprobe, and the spectra processed using the Topspin software (Bruker version 3.2). ^1^H NMR spectra were recorded at 298 K (25°C) using the Bruker-supplied “zgesgp” pulse sequence with 256 transients digitized into 32 K data points each, with a spectral width of 9615 Hz. Each spectrum was manually phased and baseline corrected, and an exponential line-broadening function (EM) of 0.5 Hz was applied.

### 2.7. Testing for Other Sources of Fatty Acids as Control Experiments

Cell culture media before and after cell growth was subjected to the entire extraction process. Additionally, a solvent blank, with no media or cells present, was also analyzed to ensure that solvents like CHCl_3_ were not contaminated with fatty acids or other impurities. Media and solvent were derivatized and analyzed by GC-MS using the same method as used for the cells. A further test on BF_3_-MeOH alone was also carried out. No fatty acids could be detected in media, solvent, or derivatization agent. As an additional control, cell extracts, solvents, and media were derivatized with 1-(trimethylsilyl)imidazole (TMSi), which is another common reagent for derivatizing fatty acids for analysis by GC-MS. These experiments also showed that fatty acids were only recovered from cell extracts. Finally, repeated analysis of different cells cultured at different times and analyzed months apart produced similar results.

## 3. Results

To assess the fatty acid content of representative archaeal organisms, hydrophobic small molecules from cultures of* S. solfataricus*,* I. hospitalis*, and* I. hospitalis-N. equitans* cocultures were extracted from cell pellets, derivatized, and analyzed using GC-MS. A mixture of chloroform and methanol was used to extract hydrophobic small molecules, using a modified Bligh-Dyer method, in which hydrophobic small molecules such as fatty acids are readily soluble in the organic phase [33]. Prior to chromatographic separation, molecules were treated with BF_3_ in methanol (BF_3_-MeOH) which reacts with electron-rich oxygen atoms of carboxylic acid groups, chemically modifying them by attaching a methyl group. Methyl group addition decreases the polarity and boiling point of the molecules, facilitating analysis by GC-MS. In addition to analyzing cell extracts, a mixture of fatty acid standards with acyl chains ranging from 14 to 24 carbons was also derivatized with BF_3_-MeOH. In order to determine the identity of the molecules, retention time and mass spectral patterns of standards and samples were matched with reference spectra accessible from the NIST database [34].

**Figure 2:**
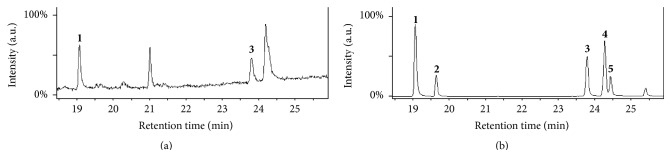
GC-MS analysis of fatty acids from cell extracts of* S. solfataricus*. Total Ion Chromatograms (TICs) of fatty acids are shown for* S. solfataricus* cell extracts (a) and lipid standard (b) as analyzed by GC-MS. Two lipids were positively identified from this analysis, matching gas chromatogram retention times and fragmentation patterns of known standards from the NIST database. GC peaks are labeled as follows:** 1**, hexadecanoic acid (C16:0);** 2**, hexadecenoic acid (C16:1);** 3**, octadecanoic acid (C18:0);** 4**, octadecenoic acid (C18:1); and** 5**, octadecadienoic acid (C18:2). The unlabeled peaks could not be positively identified but are believed to be due to instrument or solvent background based on NIST database searches.

Predicted to contain a full set of genes encoding enzymes capable of metabolizing fatty acids, we began our search for fatty acids in the aerobic organism* S. solfataricus*. Metabolite extracts from* S. solfataricus* cellstreated with BF_3_-MeOH and analyzed using GC-MS during an untargeted screen revealed MS spectral peaks with fragmentation patterns that displayed the characteristic spacing of 14 amu, typical of hydrocarbon chains [35]. Subsequent GC-MS analysis of the fatty acid standard using the same conditions yielded retention time and MS fragmentation patterns matching those for hexadecanoic acid (C16:0) and octadecanoic acid (C18:0) (Figure 2). Based on characteristic MS fragmentation patterns, several unsaturated fatty acids were also observed, but due to low signal intensity, these could not be confirmed with high confidence. This is consistent with a recently published genomic analysis, which predicted that* S. solfataricus* has genes that code for enzymes capable of metabolizing fatty acids [21]. Given that* S. solfataricus* was cultured in an undefined media (yeast extract), in-depth analyses of cell culture media and all reagents were performed. In all cases, these were negative for fatty acids, indicating that* S. solfataricus* was the origin of the fatty acids observed by GC-MS.

**Figure 3:**
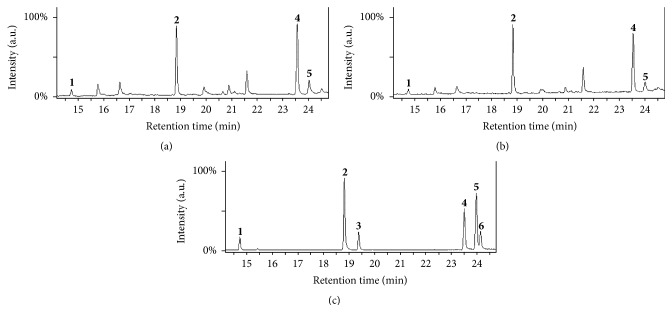
GC-MS analysis of fatty acids from cell extracts of* I. hospitalis* and* I. hospitalis-N. equitans* coculture. Total Ion Chromatograms (TICs) of fatty acids present in* I. hospitalis* cell extracts (a),* I. hospitalis-N. equitans* coculturecell extraction (b), and lipid standard (c) as analyzed by GC-MS. Four lipids were positively identified from this analysis, matching retention times of known standards and fragmentation patterns in the NIST database. GC peaks are labeled as follows:** 1**, tetradecanoic acid (C14:0);** 2**, hexadecanoic acid (C16:0);** 3**, hexadecenoic acid (C16:1);** 4**, octadecanoic acid (C18:0);** 5**, octadecenoic acid (C18:1); and** 6**, octadecadienoic acid (C18:2).

Expanding our search for fatty acids in archaeal organisms, we turned our efforts to* I. hospitalis*, a hyperthermophilic anaerobe.* I. hospitalis* was predicted to have an incomplete set of enzymes needed to carry out fatty acid metabolism, yet multiple fatty acids were positively identified based on retention time and fragmentation patterns of known standards, including tetradecanoic acid (C14:0), C16:0, C18:0, and 9-octadecenoic acid (C18:1) (Figure 3). Several other unsaturated fatty acids such as C18:2 (9,12-octadecadienoic acid) and C18:3 (9,12,15-octadecatrienoic acid) were found in low abundance but could not be confirmed with a high confidence. The confirmed presence of C18:1 indicates that* I. hospitalis* can produce unsaturated fatty acids, suggesting that it can also synthesize C18:2 and C18:3 fatty acids, a finding which is consistent with a previous study demonstrating the presence of unsaturated fatty acids in* P. furiosus* [20].* I. hospitalis* grows on a defined minimal media consisting of inorganic salts, with no yeast extract or acetate present and control experiments on media did not show the presence of fatty acids, again supporting that these fatty acids originated from the organisms [32, 36].

When* I. hospitalis* was grown in coculture with* N. equitans*, similar fatty acids were observed by GC-MS (Figure 3(b)). Previously, we have shown that when* I. hospitalis* is cocultured with* N. equitans*, an energy “tax” is imposed on* I. hospitalis* [28]. The fatty acids identified here could be a valuable energy source if available for *β*-oxidation, as the acetyl-CoA produced could be shuttled to the TCA cycle and ETS for ATP production. For the* I. hospitalis* studies, the data was collected semiquantitatively, using equal cell mass of* I. hospitalis* alone and* I. hospitalis-N. equitans* coculture. Resulting GC-MS analysis of the fatty acid contents of these different cell cultures reveals a slight decrease in fatty acid content when* N. equitans* is grown in coculture, suggesting that fatty acids are used as a source of energy to promote the growth of* N. equitans*.

**Figure 4:**
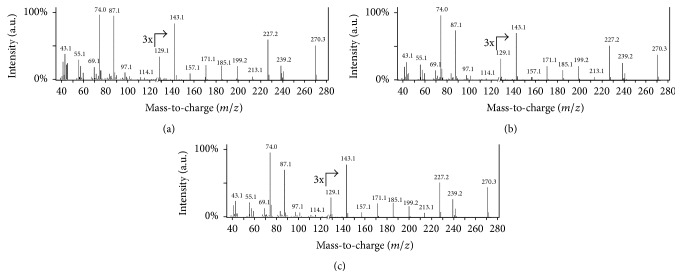
Comparison of the fragmentation patterns of fatty acids from cell extracts and standards. Representative mass spectrum for hexadecanoic acid (C16:0) from* S. solfataricus* cell extract (a),* I. hospitalis-N. equitans* coculturecell extract (b), and C16:0 fatty acid standard (c). Low and high mass fragments match intensity with the C16:0 standard, indicating that this fatty acid is present in the cell extract of these archaeal organisms. The region above 120* m/z* has been expanded 3-fold on the *y*-axis as indicated by the arrow to better visualize these low intensity ions.

To positively identify fatty acid species, fragment ions generated in the GC-MS from putative fatty acid IDs were analyzed and compared with referenced spectra of standards. When identifying fatty acids, higher mass (*m/z*) fragments are important for establishing the length of the acyl chains, while smaller mass fragments help to identify unbranched hydrocarbon chains, as well as to determine if there are unsaturated carbon-carbon bonds in the acyl chain. Characteristic features arise from each unique fatty acid, making it possible to positively identify C16:0 in extracts from all three cultures (Figure 4). Control experiments validated these findings as no fatty acid contaminants were found in solvent, media, or derivatization agent solutions (data not shown). Confirmation of fatty acids was also accomplished by matching retention times of known standards with elution peaks arising from cell culture extracts (Figures 2 and 3). A summary of the fatty acids found in the cell extracts is shown in Table 1, including fatty acids that could not be unambiguously confirmed due to their low abundance.

**Figure 5:**
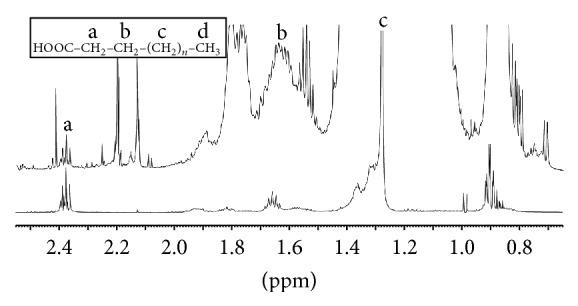
NMR analysis of fatty acids from cell extracts. 1D ^1^H NMR spectra of* S. solfataricus* cell extracts (top trace) and lipid standard (bottom trace). A general chemical formula of a saturated fatty acid is illustrated above the top NMR spectrum, with four types of hydrogen atoms experiencing different chemical environments labelled as a, b, c, and d. The characteristic chemical shifts of these chemically distinct hydrogens are assigned correspondingly to specific NMR signals in the top trace NMR spectrum.

In general, NMR is highly complementary to mass spectrometry for metabolite analysis and provides an additional level of confidence when analyzing small molecule samples, such as fatty acids [37, 38]. Lipids extracted from* S. solfataricus* were analyzed using NMR and revealed the presence of both fatty alcohols (archaeol) and fatty acids. Metabolites extracted from* S. solfataricus* were resuspended in CDCl_3_ for NMR analysis. A mixture containing 21 fatty acid standards was also analyzed in CDCl_3_ to yield a reference spectrum for the NMR lipid analysis of the cell extracts. One-dimensional (1D) ^1^H spectra recorded on* S. solfataricus* cell extract samples and fatty acid standards revealed a number of NMR signals characteristic of fatty acids (Figure 5 and Supplementary Figure 1 in Supplementary Material available online at http://dx.doi.org/10.1155/2015/472726). Analysis of* I. hospitalis* and* I. hospitalis-N. equitans* cell extracts could not be performed by NMR due to the limited amount of cell mass provided by these cell cultures.

Analysis by 1D ^1^H NMR revealed a number of spectral matches between the cellular extract and the fatty acid standard, providing further evidence that fatty acids are present in Archaea (Figure 5). A broad NMR signal appearing around 1.25 ppm (^1^H chemical shift scale) originates from methylene protons attached to the carbon chain of the fatty acid molecules. The broadness of the resonance likely corresponds to slight differences in ^1^H chemical shifts between methylene protons originating from fatty acid carbon chains of different lengths. Additional ^1^H resonances at 1.75 ppm and 2.40 ppm arise from protons attached to the *β* and *α* carbons (protons labeled “b and a” in Figure 5), whose signals are split into a multiplet and triplet, respectively (Figure 5). Protons from the methyl group at the omega end of the fatty acid chain give rise to an NMR signal at 0.90 ppm, which is split into a multiplet pattern (see Figure 5, protons labeled “d”). In the NMR spectra of the fatty acid standard mixture, a number of unsaturated fatty acids contribute to a ^1^H signal at 4.00 ppm. This same signal is observed in the ^1^H NMR spectrum of the* S. solfataricus* cell extract, although the signal is shifted slightly upfield to a ^1^H chemical shift position of 3.80 ppm. An additional, albeit weak, ^1^H signal arising from protons attached to the carbon-carbon double bond of unsaturated fatty acids is observed at a ^1^H chemical shift value of approximately 5.50 ppm.

The splitting patterns observed in the ^1^H NMR spectra are consistent with fatty acid spectral patterns, validating the positive identification of fatty acids in these two archaeal organisms by mass spectrometry. In the ^1^H NMR spectrum of the* S. solfataricus* cell extracts, the ^1^H resonance at 1.25 ppm, arising from aliphatic protons, is much broader than the corresponding signal observed in the ^1^H NMR spectrum of the fatty acid standard mixture. This broadening is attributed to the presence of archaeol lipids known to be present in archaeal organisms. Due to their large size, archaeol lipids cannot be detected by GC-MS; thus, GC-MS could not be used to validate the NMR results in this case.

## 4. Discussion

The role of lipids in biology is highly diversified. Lipids are foundational components of biological membranes, are stable and efficient energy storage molecules, and can also function as effective signaling molecules. Their presence in archaeal cells, even in small quantities, is noteworthy. This work demonstrates that two hyperthermophilic Archaea,* S. solfataricus* and* I. hospitalis*, contain fatty acids. It is well established that these organisms use ether-linked fatty lipids (fatty alcohol-based) molecules as membrane components, due to the increased thermal stability imparted by these lipids on membrane structures. Recent genomic analysis of archaeal species suggested that, in addition to fatty alcohols, a number of extremophiles possess genes that could encode proteins capable of synthesizing and catabolizing fatty acids [21]. Using a combination of GC-MS and NMR, we have clearly demonstrated the presence of fatty acids in both organisms. These data provide evidence that these two organisms, with very different metabolic requirements and environments (e.g., aerobic versus anaerobic), have the enzymatic machinery necessary for fatty acid metabolism.

In* S. solfataricus*, saturated fatty acids were identified in relatively high amounts by matching retention time and fragmentation patterns using GC-MS and NMR. Previous analysis of the membrane lipid content of* S. solfataricus* did not reveal the presence of any fatty acids but instead showed the isoprenoid-based ether-linked lipids [28]. The present study using GC-MS did not identify fatty alcohols, because the approach used to generate FAMES does not readily hydrolyze the ether linkages of archaeol lipids. Additionally, the intact archaeol lipids themselves are too large for GC-MS analysis given that their molecular weight is well above the practical working range of gas chromatography and electron ionization used for GC-MS studies. The presence of fatty acids in* S. solfataricus* indicates that this organism possesses the enzymatic machinery necessary for their biosynthesis and strongly suggests that these lipids can be catabolized as an energy source.

As strict anaerobes,* I. hospitalis* and* N. equitans* offer an entirely different perspective on fatty acid metabolism in the archaeal domain. Growing under highly reducing conditions and high pressure, with CO_2_ as the sole carbon source, we expect energy to be derived from H_2_ and elemental sulfur. These two organisms are prototypical of a minimalistic lifestyle. This carries over to their genomes which contain a reduced set of genes and were not listed among the Archaea predicted to contain all of the necessary protein machinery for fatty acid metabolism. Therefore, our identification of fatty acids in* I. hospitalis and I. hospitalis-N. equitans* cocultures was surprising and supports the idea that a nonorthologous enzyme may be present which has acyl-CoA dehydrogenase (ACD) functionality [21]. In addition to saturated fatty acids, as observed in* S. solfataricus*, unsaturated molecular species were also identified. Unsaturated fatty acids require additional enzymatic machinery for their synthesis including desaturases to generate carbon-carbon double bonds.

Together, the results of the GC-MS and NMR analysis of lipid extracts confirm the presence of fatty acids in Archaea. Our finding is consistent with genomic evidence that the metabolic pathways needed to produce and consume fatty acids exist in* S. solfataricus* and indicates that the missing enzyme from* I. hospitalis* (ACD or a homolog as suggested by Dibrova et al.) must be present [21]. Inspired by our biochemical data, a position-specific BLAST search of the genome of* I. hospitalis* was conducted to search for* I. hospitalis* enzymes matching acyl-CoA dehydrogenases from other Archaea. No sequence matches or other enzymes with predicted EC numbers capable of carrying out this reaction were found. This finding supports the idea proposed by Dibrova et al. that an alternative ACD enzyme may be present in* I. hospitalis*. Furthermore, genomic analysis of* I. hospitalis* and* S. solfataricus* failed to connect fatty acid metabolism with membrane systems involved with electron transport such as cytochromes and rhodopsins as described in other systems [21].

Given that this organism lives in an energy-limited environment and processes a minimal genome, it is likely that* I. hospitalis* has evolved an enzyme capable of carrying out multiple biosynthetic reactions, including that for the metabolism of fatty acids. An intriguing hypothesis is whether* I. hospitalis* contains an enzyme system engineered without catalytic bias toward biosynthesis or *β*-oxidation. Such a system could use the concentration of reactants and products (acyl-CoA and fatty acids, resp.) to regulate the anabolic versus catabolic direction of fatty acid metabolism. This concept is consistent with ideas put forth by Dibrova et al. in their proposed mechanisms of fatty acid metabolism [21].

The identification of a new potential source of metabolic energy in these hyperthermophilic acidophiles raises questions about their biological role. Given that fatty acids similar to those found in this study have been found in other archaeal organisms, it is likely that they would be used as an energy source, as fatty acids are storable, generate twice the energy of carbohydrates by weight (9 kcal/g versus 4 kcal/g, resp.), and could be a very important energy source for archaeal organisms which often inhabit energy-depleted environments [39]. Unlike* S. solfataricus* which lives in an aerobic environment,* I. hospitalis* requires an anaerobic environment and likely makes use of a sulfate coupled reduction to perform fatty acid oxidation [30]. Additionally,* I. hospitalis* is an obligate chemolithoautotrophic organism, thought to survive solely on the reduction of sulfur for energy production. With the identification of enzymes capable of carrying out fatty acid metabolism and fatty acid molecules themselves, it is worth reexamining the metabolic capacity and potential value of fatty acids to organisms that live in harsh, energy-limited environments.

## Supplementary Material

Supplementary Figure 1. NMR analysis of fatty acids from cell extracts. 1D 1H NMR spectra of S. solfataricus cell extracts (A) and lipid standard (B). The insert in panel A corresponds to an expanded view of the 1.5 to 2.5 ppm spectral region of the 1D 1H NMR spectrum. The insert in panel B displays the generic chemical formula of a saturated fatty acid, with four distinct 1H chemical environments labelled as a, b, c, and d. The characteristic chemical shifts of these 1H are labeled correspondingly in both the 1D 1H NMR spectra of the S. solfataricus cell extract shown in panel A and the lipid standard sample shown in panel B.

## Figures and Tables

**Table 1:** Fatty acids identified in archaeal cell extracts of *S. solfataricus*, *I. hospitalis*, and *I. hospitalis-N. equitans*.

Fatty acid	Cell culture		
	*S*. *solfataricus*	*I*. *hospitalis*	*I*. *hospitalis*-*N*. *equitans*
Tetradecanoic acid (C14:0)		X	X
Hexadecanoic acid (C16:0)	X	X	X
9-Hexadecenoic acid (C16:1)	X^*∗*^	X	X
Octadecanoic acid (C18:0)	X	X	X
9-Octadecenoic acid (C18:1)	X^*∗*^	X	X
9,12-Octadecadienoic acid (C18:2)		X	X
9,12,15-Octadecatrienoic acid (C18:3)		X^*∗*^	X^*∗*^
Tetracosanoic acid (C24:0)		X^*∗*^	X^*∗*^
Hexacosanoic acid (C26:0)		X^*∗*^	X^*∗*^

^*∗*^denotes trace amounts, not able to confirm unambiguously cocultures.
