# Supplementary material for: Characterization of Fatty Acids in Crenarchaeota by GC-MS and NMR
Source: Archaea. 2015 Dec 31;2015:472726. doi: 10.1155/2015/472726 (PMC4736080; doi:10.1155/2015/472726)
Supplement: Supplementary file 1 — Supplementary Figure 1. NMR analysis of fatty acids from cell extracts. 1D 1H NMR spectra of S. solfataricus cell extracts (A) and lipid standard (B). The insert in panel A corresponds to an expanded view of the 1.5 to 2.5 ppm spectral region of the 1D 1H NMR spectrum. The insert in panel B displays the generic chemical formula of a saturated fatty acid, with four distinct 1H chemical environments labelled as a, b, c, and d. The characteristic chemical shifts of these 1H are labeled correspondingly in both the 1D 1H NMR spectra of the S. solfataricus cell extract shown in panel A and the lipid standard sample shown in panel B. [file 472726.f1.pdf]

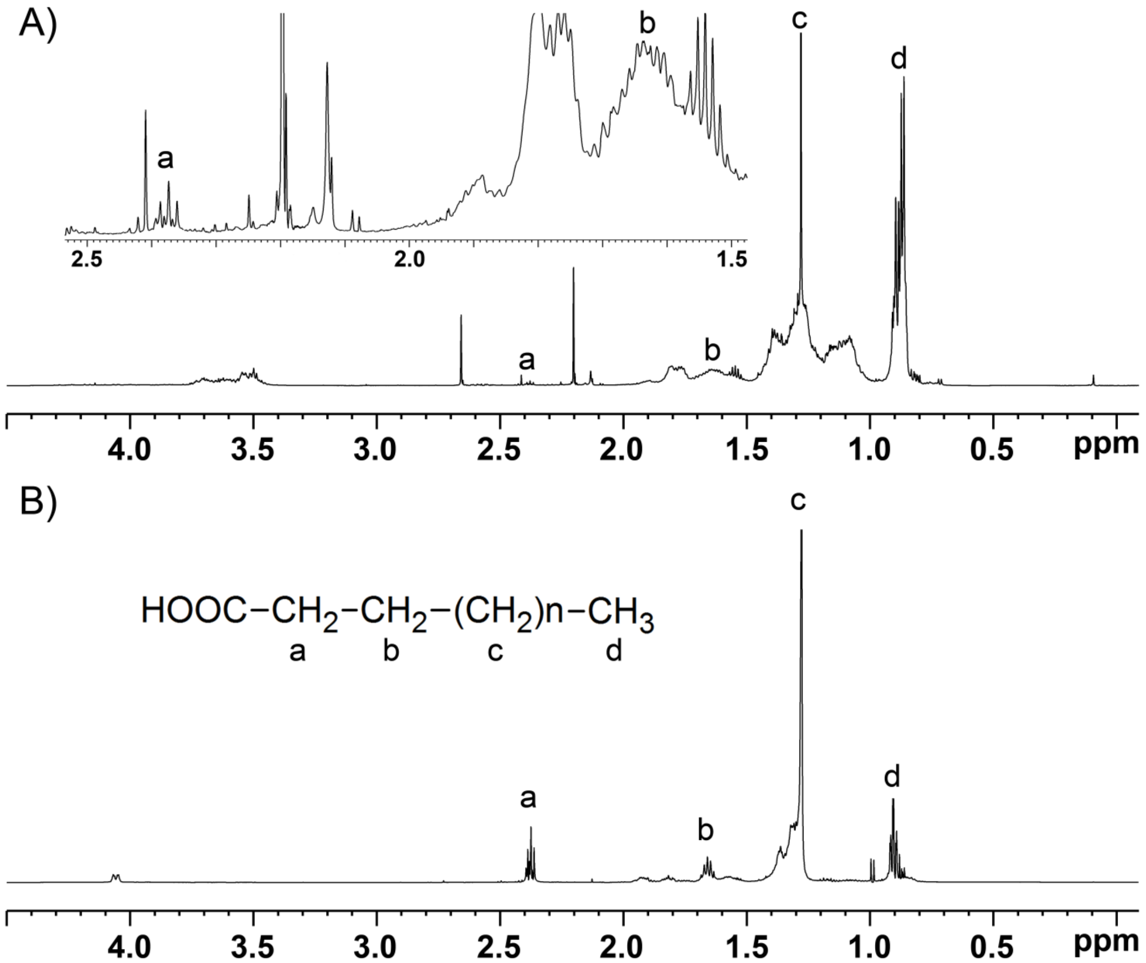

Supplementary Figure 1. NMR analysis of fatty acids from cell extracts. 1D ^{1}H NMR spectra of *S. solfataricus* cell extracts (A) and lipid standard (B). The insert in panel A corresponds to an expanded view of the 1.5 to 2.5 ppm spectral region of the 1D ^{1}H NMR spectrum. The insert in panel B displays the generic chemical formula of a saturated fatty acid, with four distinct ^{1}H chemical environments labelled as a, b, c, and d. The characteristic chemical shifts of these ^{1}H are labeled correspondingly in both the 1D ^{1}H NMR spectra of the *S. solfataricus* cell extract shown in panel A and the lipid standard sample shown in panel B.
